# Seminal 5′tRF-Glu-CTC as a Biomarker for Oligozoospermia and ART Outcome Prediction

**DOI:** 10.1186/s12610-025-00283-0

**Published:** 2025-09-01

**Authors:** Neslihan Hekim, Sezgin Gunes, Bulent Ayas, Elzem Nisa Alkan

**Affiliations:** 1 Department of Medical Biology, Faculty of Medicine, Ondokuz Mayis University, Samsun, Turkey; 2 Department of Molecular Medicine, Graduate Institute, Ondokuz Mayis University, Samsun, Turkey; 3 Department of Histology and Embryology, Faculty of Medicine, Ondokuz Mayis University, Samsun, Turkey

**Keywords:** Oligozoospermia, Sperm DNA fragmentation, tRNA-derived fragments, Assisted reproductive technology, 5'tRF-Glu-CTC

## Abstract

**Background:**

Epigenetic mechanisms influencing sperm production and function are closely linked to male infertility. Among these, tRNA-derived fragments have recently been identified as important modulators of cellular stress responses and gene expression. The purpose of this study was evaluate the potential role of 5'tRF-Glu-CTC in the context of assisted reproductive technologies (ART) outcomes by comparing its differential expression in the seminal plasma of oligozoospermic and normozoospermic infertile men.

**Results:**

Total RNA was extracted from seminal plasma, and 5'tRF-Glu-CTC expression was measured by qRT-PCR. Sperm DNA fragmentation index (DFI) was assessed via the TUNEL assay. The results revealed significantly elevated 5'tRF-Glu-CTC expression in the oligozoospermic group compared to the normozoospermic group (15.58 ± 4.34 vs. 12.53 ± 4.99; fold change: 1.692; P = 0.024). Sperm DFI showed a significant negative correlation with both the progressive motile sperm (ρ = -0.537, P = 0.015) and the total progressive motile sperm count (ρ = -0.509, P = 0.026). However no significant differences in DFI was observed between the oligozoospermic and control groups (P > 0.05). Analysis of ART outcomes revealed that male partners of couples who achieved pregnancy had lower mean 5'tRF-Glu-CTC expression and DFI, although these differences did not reach statistical significance (P > 0.05).

**Conclusions:**

These results suggest that 5'tRF-Glu-CTC might be serve as a potential biomarker and could play a role in the pathophysiology of oligozoospermia.

## Résumé

**Contexte:**

Les mécanismes épigénétiques influençant la production et la fonction des spermatozoïdes sont étroitement liés à l’infertilité masculine. Parmi ceux-ci, des fragments dérivés de l’ARNt ont récemment été identifiés comme d’importants modulateurs de réponses au stress cellulaire et de l’expression des gènes. Le but de cette étude était d’évaluer le rôle potentiel de la 5'tRF-Glu-CTC dans le contexte des résultats des techniques de procréation assistée (ART) en comparant son expression différentielle dans le plasma séminal d’hommes infertiles présentant soit une oligozoospermie soit une normozoospermie.

**Résultats:**

L’ARN total a été extrait du plasma séminal, et l’expression de 5'tRF-Glu-CTC a été mesurée par qRT-PCR. L’indice de fragmentation de l’ADN des spermatozoïdes (DFI) a été évalué par le test TUNEL. Les résultats ont révélé une expression significativement élevée de 5'tRF-Glu-CTC dans le groupe oligozoospermique par rapport au groupe normozoospermique (15,58 ± 4,34 vs 12,53 ± 4,99; facteur de changement: 1,692; P = 0,024). Le DFI des spermatozoïdes a montré une corrélation négative significative avec les spermatozoïdes mobiles progressifs (r = -0,537, P = 0,015) et le nombre total de spermatozoïdes mobiles progressifs (r = -0,509, P = 0,026). Cependant, aucune différence significative n’a été observée entre les groupes oligozoospermique et normozoospermique (P > 0,05). L’analyse des résultats de l’ART a révélé que les partenaires masculins des couples qui ont obtenu une grossesse avaient une expression moyenne plus faible de 5'tRF-Glu-CTC et de DFI, bien que ces différences n’aient pas atteint la signification statistique (P > 0,05).

**Conclusions:**

Ces résultats suggèrent que le 5'tRF-Glu-CTC pourrait servir de biomarqueur potentiel et pourrait jouer un rôle dans la physiopathologie de l’oligozoospermie.

**Mots-clés:**

Oligozoospermie ; Fragmentation de l’ADN des Spermatozoïdes ; Fragments dérivés de l’ARNt ; Technique de Procréation assistée ; 5'tRF-Glu-CTC.

## Introduction

Male fertility is predominantly regulated by epigenetic mechanisms such as DNA methylation, histone modifications, and a variety of noncoding RNAs that affect sperm production and function [1]. While infertility may impair the natural transmission of genetic and epigenetic traits, assisted reproductive technologies (ART) present the possibility of passing traits to the next generation.

Genome-wide association studies and familial inheritance analyses often fall short in identifying the underlying genetic contributors due to the complex nature of male infertility, including its specificity and heterogeneity, the unique characteristics of spermatozoa compared to somatic cells, the inherent heterogeneity of sperm within semen, and the fully differentiated state of mature sperm [1]. Moreover, understanding the influence of environmental factors on these epigenetic processes is critical for establishing effective preventative and treatment strategies. These challenges highlight the importance of multidisciplinary investigative approaches to uncover the molecular pathways involved in male infertility.

Following transcription by RNA polymerase III, transfer RNA precursors (pre-tRNAs) undergo several key processing steps, including endonucleolytic and exonucleolytic elimination of the 5′ leader and the 3′ trailer sequences, post-transcriptional addition of CCA trinucleotide to the 3′ end, and incorporation of a guanosine residue to the 5′ end of tRNA^His^, and enzymatic splicing of the introns in certain types of tRNAs [2, 3]. Recent findings have revealed that these mature and precursor tRNAs can be enzymatically cleaved to generate various tRNA-derived fragments (tRFs), which represent a newly recognized layer of epigenetic regulation, with underlying mechanisms discovered in recent years [2, 4]. tRFs contain a distinct subgroup of regulatory non-coding RNA groups that play role gene expression regulation and also in intracellular non-canonical functions. These molecules are involved in a range of physiological and pathological processes [2, 3]. In response to environmental stress, certain tRFs may bind to cytochrome C, preventing apoptosis by lowering caspase 3 activation [4]. tRFs have been found to bind proteins from the Argonaute family or interact directly with the 3'untranslated region (UTR) of target mRNA to regulate the expression of a gene [3]. Furthermore, tRFs have been identified as important mediators of the genes expression regulation and stress responses [5]. Given these roles, tRFs hold significant potential as biomarkers for diagnosis and as targets for therapeutic interventions.

tRF profiles may also be serve as valuable biomarkers for assessing sperm quality and predicting ART success. In the present study the expression differences of 5’tRF-Glu-CTC between oligospermic and normozoospermic infertile men were examined. Furthermore, the possible involvement of 5'tRF-Glu-CTC in achieving successful ART outcomes has been investigated.

## Patients and methods

### Participants and sample collection

Male partners of couples who applied to Ondokuz Mayis University (OMU) IVF Center with infertility complaints between September 2023 and February 2025 were included to the study. Men experiencing infertility due entirely to male causes were included, as long as their female partner was healthy and had a normal obstetric evaluation. All participants were between the ages of 18 and 40 and had no known genetic abnormalities. Couples were excluded if female partners had any contributing infertility factors, such as being under 18 or over 35 years old, having irregular menstrual cycles, evidence of anovulation, tubal obstruction, or a history of significant medical conditions (e.g., cancer, heart disease, or cirrhosis, history of polycystic ovary syndrome) or prior sterilization procedures (e.g., tubal ligation). Male participants who were undergoing treatment or taking medications known to impact reproductive function or metabolism (such as inflammatory bowel disease, antihypertensives (calcium channel blockers), testosterone and anabolic steroids, chemotherapy within the past year, selective estrogen receptor modulators (SERMs), aromatase inhibitors, gonadotropins, or obstructive, non-obstructive azoospermia, or severe oligospermia with other confounding conditions) were not included. Additional exclusion criteria included untreated hypothyroidism and uncontrolled diabetes mellitus for infertile men. Male partners of couples undergoing ART treatment were included in the study based on their semen analysis results. Randomization and group allocation were performed according to sperm concentration, and participants were divided into two groups: oligozoospermic (sperm concentration < 16 million/mL) and normozoospermic (sperm concentration ≥ 16 million/mL). All participants were informed about the purpose and procedures and were included in the study. Participants were enrolled if they gave written informed permission. Every participant filled out a comprehensive information form that included contact information, the results of a semen analysis, their overall health, and any history of infertility in their family or personal life.

### Isolation of tRFs and cDNA conversion

The seminal plasma was separated from the sperm pellet by centrifuging the semen samples for five minutes at 600x*g*. Total RNA, including tRNA-derived fragments (tRFs), was isolated from seminal plasma using the SanPrep Column microRNA Miniprep Kit (Biobasic, Cat. No. SK8811). Concentrations and purity of the RNA were assessed using a microplate spectrophotometer (Multiscan GO, Thermo Scientific, Finland). Reverse trancription (RT) was carried out by miRNA All-In-One cDNA Synthesis Kit (ABM, Cat. No. G898), employing stem-loop oligomer probes. Each reaction consisted of 10 μl of 2 × miRNA cDNA Synthesis SuperMix, 200 ng of RNA template, enzyme mix, stem-loop oligomer probes and nuclease-free H_2_O, making up a total volume of 20 μl. Table 1 lists the primers and probes used in RT and qRT-PCR assays.

**Table 1:** Primers and stem-loop probes used in reverse transcription and qRT-PCR assays

Parameter	Probes and Primers
5’tRF-Glu-CTC	Probe: 5’-GTCTCCTCTGGTGCAGGGTCCGAGGTATTCGCACCAGAGGAGACCGTGCCG-3’
	Forward 5’-GGCGGTCCCTGGTGGTCTAGTGGTTAGGATT-3’
miR-320 a/b	Probe: 5’-GTCTCCTCTGGTGCAGGGTCCGAGGTATTCGCACCAGAGGAGACTYGCCC-3’
	Forward 5’-GGCGGAAAAGCTGGGTTG AGA-3’
Universal	Reverse 5’-TGGTGCAGGGTCCGAGGTATT-3’

### tRF expression analysis with qRT-PCR

The expressions of 5’tRF-Glu-CTC was evaluated using expression primers designed specifically (Table 1). Following the synthesis of cDNA, qRT-PCR was performed using BlasTaq™ 2X qPCR MasterMix (Applied Biological Materials, Canada) on a BioRad® CFX96 Touch. Reaction mixtures consisted of 10 μl of BlasTaq™ 2X qPCR MasterMix, 0.5 μl of each 10 μM forward and reverse primers and template cDNA in a final 20 μl volume. The expression levels of 5’tRF-Glu-CTC and the reference small RNA miR-320 were measured in duplicate. Mean quantification cycle (Cq) values ​​of 5’tRF-Glu-CTC were normalized to miR-320 for each samples. Relative gene expression changes were performed using the formula 2^−ΔΔCq^ = 2^−[Sample ΔCq (Gene−Reference) −Control ΔCq (Gene−Reference)]^ [1].

### Terminal deoxynucleotidyl Transferase-Mediated dUTP Nick-End Labeling (TUNEL) for Sperm DNA fragmentation analysis

Semen pellets were fixed with 3.6% paraformaldehyde and then dropped onto poly-L-lysine coated slides using phosphate buffer containing sucrose. Following overnight incubation, the reaction was performed with the In situ Apoptosis Detection Kit (Takara Bio Inc., Shiga, Japan) in accordance with the manufacturer's instructions. During the assay, fluorescein-labeled nucleotides were incorporated at the 3'-OH ends of fragmented DNA, enabling in situ labeling. Nuclear counter staining was performed using DAPI. Spermatozoa were analyzed using a fluorescent microscope equipped with FITC and DAPI filters, and their general morphology was examined using a light microscope. At least 5 fields and a minimum of 200 cells were analyzed for each sample. Quantification was performed with ImageJ software (Version 1.54p), a Java-based image analysis tool. DNA fragmentation index (DFI) was calculated by dividing the number of spermatozoa exhibiting FITC fluorescence (which indicates DNA fragmentation) by the total number of spermatozoa sperm counted.

### Statistical analysis

The number of volunteers required for each group was calculated as 15, with a test power of 87.6% and a margin of error of 0.05, using the Minitab16.0 program. Descriptive data were presented as mean, median, standard deviation (SD) and standard error (SE). Kolmogorov–Smirnov test was carried out to assess the normality of the data distribution. Following normality testing, pairwise comparisons between groups for normally distributed and non-normally distributed data were performed using the Student's t-test and Mann–Whitney U, respectively. In analyses with more than two groups, ANOVA was used for normally distributed variables, the Kruskal–Wallis tests was applied for non-normally distributed data. Gene expression alterations were evaluated based on fold change. Correlation between the experimental outcomes and the clinical parameters of the participants was evaluated by Pearson’s correlation coefficient for parametric or Spearman’s rank correlation coefficient for non-parametric data. The statistical analysis was conducted using the SPSS v21 program (IBM software, Pointe Claire, Quebec, Canada), with P < 0.05 indicating statistical significance.

## Results

Semen analyses of the participants included in the study were evaluated according to the sixth edition of the World Health Organization (WHO) Laboratory Manual for the Examination and Processing of Human Semen [6]. The distribution of ages of participants in the two groups did not show statistically significant difference.

Following normalization of Cq values ​​obtained through qRT-PCR using miR-320 as the reference, the mean 5’tRF-Glu-CTC expression level in seminal plasma of oligozoospermic group was found to be significantly higher in the oligozoospermic group compared to the normozoospermic group. The increase in seminal expression of 5'tRF-Glu-CTC in the oligozoospermic group was 1.692-fold.

**Fig. 1:**
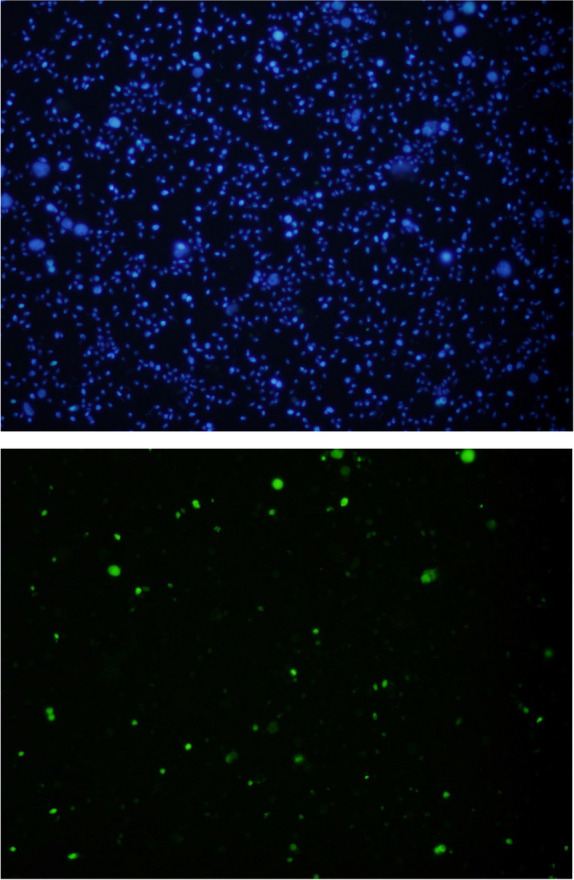
Fluorescence microscopy images obtained under DAPI (on the top) and FITC (in the middle) filters of the same field. × 40 magnification

Sperm DFI was assessed using in situ TUNEL analysis. Figure 1 shows representative fluorescence microscopy pictures for sperm DFI analysis. There was no statistically significant difference was found between these two groups in terms of sperm DFI. Sperm DNA fragmentation was not found to be correlated with 5’tRF-Glu-CTC expression. However, sperm DNA fragmentation was negatively correlated with total progressive motile sperm count, percentage of progressive motile sperm and total motility. A positive correlation was observed between sperm DNA fragmentation and the percentage of immotile sperm. Clinical data, semen parameters, sperm DFI, seminal plasma tRF expression levels for normozoospermic (n = 25) and oligozoospermic (including cases of oligoasthenoteratozoospermia and oligoasthenozoospermia) (n = 23) groups are summarized in Table 2.

**Table 2:** Clinical parameters of oligozoospermic and normozoospermic groups

Clinical Parameters	Oligozoospermic group	Normozoospermic group	P-value
	**(n = 23)**	**(n = 25)**	
	**Mean ± SD** **(Median ± SEM)**	**Mean ± SD** **(Median ± SEM)**	
Age, year	35.91 ± 5.96 (36.50 ± 1.27)	34.91 ± 4.81 (35.50 ± 1.03)	NS
Semen Volume, ml	3.10 ± 1.09 (3.00 ± 0.24)	2.78 ± 0.81 (3.00 ± 0.19)	NS
Sperm Concentration, 10^6^/ml	9.28 ± 6.66 (7.00 ± 1.49)	30.94 ± 12.14 (30.00 ± 2.94)	**0.001**
Total Sperm Count, 10^6^/ejeculate	25.55 ± 18.16 (20.00 ± 4.06)	87.18 ± 36.98 (96.00 ± 8.97)	**0.001**
Total Progressive Motile Sperm Count, 10^6^/ejeculate	6.28 ± 7.97 (2.55 ± 1.78)	35.50 ± 23.63 (36.00 ± 6.82)	**0.001**
Progressive Motile Sperm (Mot a), %	17.00 ± 14.37 (12.50 ± 3.21)	38.77 ± 16.46 (43.00 ± 4.56)	**0.001**
Non-Progressive Motile Sperm (Mot b), %	5.4500 ± 2.16 (5.00 ± 0.48)	6.00 ± 2.45 (5.00 ± 0.68)	NS
Immotile Spermatozoa, %	77.55 ± 15.25 (80.00 ± 3.41)	52.07 ± 18.80 (51.00 ± 5.02)	0.001
Motility (Mot a + b), %	22.45 ± 15,25 (20.00 ± 3.41)	53.06 ± 19.22 (52.50 ± 4.53)	**0.001**
Normal Morphology, %	3.84 ± 1.26 (4.00 ± 0.29)	6.82 ± 2.99 (5.00 ± 0.90)	**0.001**
tRF Expressions (ΔCq)	15.58 ± 4.34 (14.58 ± 0.91)	12.53 ± 4.99 (11.09 ± 1.00)	**0.024**
DFI, %	17.38 ± 15.34 (12.05 ± 4.43)	12.01 ± 8.58 (9.42 ± 2.02)	NS

Differences among the groups were analyzed using Independent-Samples Mann–Whitney U test. Statistical significance was determined as P < 0.05. Statistically significant results are indicated in bold. *NS* Non significant

### ART outcomes

Samples from the male partners of the infertile couples participating in the study were further analyzed in relation to ART outcomes (Table 3) following 5’tRF-Glu-CTC expression level and DFI assessment. For this purpose, expression and fragmentation results were compared between the subgroups stratified by the occurrence of the two pronuclei (2PN) oocyte retrieval, embryo formation and pregnancy. The mean 5’tRF-Glu-CTC expression was higher in the groups where 2PN oocytes were retrieved and embryos formed (13.09 ± 4.44 and 15.81 ± 5.38, respectively) compared to those in which these outcomes were not achieved. Additionally, both the mean 5’tRF-Glu-CTC expression (12.96 ± 2.95 and 14.87 ± 5.26) and DFI values (10.40 ± 10.56 and 16.16 ± 21.45) were lower in the group who achieved a positive pregnancy compared to those who did not. However, the mean differences did not reach statistical significance (P > 0.05). Overall, no significant correlations were observed between 5'tRF-Glu-CTC expression or sperm DFI and ART outcomes, including 2PN oocyte, embryo formation or positive pregnancy (P > 0.05).

**Table 3:** ART outcomes of participants

Patient	Group	Oocyte	Oocyte_2PN	Embryo	Transfer	Pregnancy
P 1	Normo					
P 2	Normo					
P 3	Normo	19	16	14		Positive
P4	Normo	0				
P 5	Normo	4				
P 6	Normo	10	7	7		Positive
P 7	Normo					
P 8	Normo	5	4	3	1	Positive
P 9	Normo	9	2	2	1	Negative
P 10	Normo	2	1	1	1	
P 11	Normo	22	8	8	1	
P 12	Normo	8				
P 13	Normo	9	8	5	1	
P 14	Normo	0				
P 15	Normo	6	4	4		
P 16	Normo	9	7	6	1	
P 17	Normo	4	3	3	1	
P 18	Normo	15	6	5	1	Positive
P 19	Normo	1	0	0		
P 20	Normo	12	9	9	1	Positive
P 21	Normo	18	13	13		
P 22	Normo	0				
P 23	Normo	6	5	5	1	
P 24	Normo	5	2	2	2	
P 25	Normo	3	2	2	2	
P 26	OAT/OA	31				
P 27	OAT/OA	6	2	2		
P 28	OAT/OA	7				Positive
P 29	OAT/OA	3	2	1		
P 30	OAT/OA	7	3	2	1	
P 31	OAT/OA					
P 32	OAT/OA					
P 33	OAT/OA					
P 34	OAT/OA					
P 35	OAT/OA	7				Positive
P 36	OAT/OA					
P 37	OAT/OA	16	0		0	
P 38	OAT/OA	0				
P 39	OAT/OA					
P 40	OAT/OA	1	0		0	
P 41	OAT/OA	18	8	8	1	Positive
P 42	OAT/OA	6	0	0	0	Negative
P 43	OAT/OA	5	4	4	2	
P 44	OAT/OA	9	6	5	2	
P 45	OAT/OA	27	14	14	1	
P 46	OAT/OA	8	4	4	1	Positive
P 47	OAT/OA	11	6	6	1	
P 48	OAT/OA	10	5	5	2	Negative

*2PN* two pronuclei, *Normo* Normozoospermia, *OAT* Oligoasthenoteratozoospermia, *OA* Oligoasthenospermia, *P* Participant

## Discussion

In this study, the expression of 5'tRF-Glu-CTC in seminal plasma was found to be significantly elevated in oligozoospermic individuals compared to normozoospermic men. 5'tRFs are a class of tRNA-derived small RNAs have recently been linked in epigenetic regulation of gene expression, cellular stress responses and translational repression [7, 8]. Specifically, 5'tRF-Glu-CTC has been associated with ribosomal functions and translational control, and its expression has been found to change under different pathophysiological conditions [9, 10]. Moreover, it is hypothesized that microenvironmental variations associated with oligozoospermia, such as oxidative stress and inflammation, may affect tRF expression profiles [11]. Recent research suggests that tRFs may serve as novel indicators with potential significance to sperm quality and male fertility [10, 12]. Our findings suggest that 5'tRF-Glu-CTC may contribute to the pathophysiology of oligozoospermia or serve as a molecular biomarker of this condition. However, the mechanistic basis of this association remains unclear and requires further investigation through detailed molecular and functional studies.

This study also investigated the expression levels of 5’tRF-Glu-CTC in the seminal plasma and sperm DNA fragmentation in men undergoing ART, as well as the link between these parameters and both semen analysis and ART results. Although limited literature includes several intriguing studies highlight the potential of specific tRNA-derived fragments as biomarkers, particularly in the context of male subfertility [13–16]. Sequencing and qRT-PCR analyses of seminal non-coding RNAs (ncRNAs) from infertile patients demonstrated that tRF-Val-AAC and tRF-Pro-AGG had the potential to predict ART success in men with azoospermia, outperforming traditional markers with high specificity and sensitivity such as serum follicle-stimulating hormone (FSH) [16]. Other research has also shown that tRF-Gly-GCC and tRF-Glu-CTC are good predictors of effective sperm retrieval with microdissection testicular sperm extraction (micro-TESE) in patients with non‐obstructive azoospermia (NOA) [15].

Another study reported that when sperm from normozoospermic males was used to fertilized oocytes from young donors, the expression levels of sperm-derived tRFs specifically 5'tRF-Asp-GTC, 5'tRF-Phe-GAA, and 3'tRF-Ser-GCA were differed significantly between groups with high and low blastocyst formation rates [13]. Although the biological role of 5’tRF-Glu-CTC is not fully understood, emerging evidence suggests that tRF may alter spermatozoa function and thus male fertility. Furthermore, this tRF has been proposed to be associated with sperm DNA integrity and hormonal regulation [14].

A recent study discovered that the expression levels of 5'tRF-Glu-CTC, 5'tRF-Lys-CTT, and 5'tRF-Gly-GCC in seminal plasma were significantly higher in normospemic infertile males with recurrent ART failures [14]. Notably, higher 5’tRF-Glu-CTC levels were observed in men whose partners were unable to achieve pregnancy and these findings are consistent with the present study. The researchers also stated that there was no significant difference in sperm DFIs between the groups, but they discovered an unexpected negative correlation between sperm DFI and seminal 5'tRF-Glu-CTC levels [14]. In contrast, the present study did not find a correlation between seminal expression of 5’tRF-Glu-CTC and DNA fragmentation in sperm. However, among couples who achieved pregnancy, both 5’tRF-Glu-CTC expression and sperm DNA fragmentation levels were found to be lower than expected. Although this difference was not statistically significant, it may indicate a biologically relevant trend. The increase in the expression of seminal 5'tRF-Glu-CTC in oligozoospermic men becomes more significant when considered alongside with sperm DNA integrity. Increased sperm DNA fragmentation rates have frequently been documented in oligozoospermic individuals and this condition has been found to be strongly associated with male infertility [17]. DNA fragmentation can result from processes such as increased oxidative stress, inflammation or apoptosis within the testicular microenvironment and may also impact epigenetic regulations during spermatid maturation [18, 19]. Recent studies have revealed that specific tRFs in spermatozoa may play a regulatory role in maintaining DNA integrity, particularly during spermatogenic maturation [10, 12]. In this context, reduced 5'tRF-Glu-CTC levels may serve as an indicator of a more stable sperm DNA architecture.

This study has several limitations that should be considered when interpreting the findings. First, the sample size was relatively small, particularly in subgroup analyses related to ART outcomes, which may limit the generalizability of the results. Second, while the expression level of 5’tRF-Glu-CTC was assessed in seminal plasma, functional validation studies were not performed to elucidate its precise molecular role in sperm physiology or fertilization processes. Finally, although sperm DNA fragmentation was evaluated, other critical markers of oxidative stress and epigenetic modifications were not assessed, which could provide a more comprehensive understanding of the underlying mechanisms.

## Conclusions

The findings demonstrate that seminal plasma levels of 5’tRF-Glu-CTC are significantly elevated in oligozoospermic men and may be associated with impaired sperm DNA integrity and suboptimal ART outcomes. These results highlight the potential of 5’tRF-Glu-CTC as a novel biomarker for male infertility, particularly in the context of oligozoospermia. Clinically, this tRF could aid in identifying patients at risk of poor reproductive outcomes and help guide personalized treatment strategies. Future studies with larger cohorts, functional validation experiments, and longitudinal ART outcome tracking are warranted to better understand the mechanistic role of 5’tRF-Glu-CTC in male fertility and to evaluate its potential as a predictive or therapeutic target.

## Data Availability

The data supporting the findings of this study are available within the paper and are also available from the corresponding author.

## Abbreviations

ANOVA: Analysis of Variance
ART: Assisted Reproductive Technologies
DFI: DNA fragmentation index
FSH: Follicle-stimulating hormone
ncRNA: Non-Coding RNA
NOA: Non‐obstructive azoospermia
UTR: Untranslated region
PBS: Phosphate Buffered Saline
SERM: Selective Estrogen Receptor Modulators
tRFs: TRNA-Derived Fragments
tRNA: Transfer RNA
TUNEL: Terminal Deoxynucleotidyl Transferase-Mediated dUTP Nick-End Labeling
qRT-PCR: Quantitative Reverse Transcription Polymerase Chain Reaction
WHO: World Health Organization
